# Pagetoid Dyskeratosis of the Male Genitalia: Case Report and Review

**DOI:** 10.7759/cureus.2727

**Published:** 2018-06-01

**Authors:** Tyler Werbel, Philip R Cohen

**Affiliations:** 1 School of Medicine, University of California, San Diego, San Diego, USA; 2 Dermatologist, San Diego Family Dermatology, National City, USA

**Keywords:** cell, clear, dyskeratosis, genitalia, pagetoid, penile, penis, prepuce, scrotum, shaft

## Abstract

Pagetoid dyskeratosis is a benign incidental pathologic finding that has been reported in many distinct skin lesions on various locations of the body. A man who had pagetoid dyskeratosis within lesions of the penile shaft is described and similar cases of pagetoid dyskeratosis in lesions of the male genitalia are reviewed. The patient was a 26-year-old healthy man who developed several asymptomatic penile papules that were refractory to topical imiquimod 5% cream and cryotherapy. Snip biopsies were performed and microscopic examination revealed pagetoid dyskeratosis. PubMed was searched for the following terms: cell, clear, dyskeratosis, genitalia, pagetoid, penile, penis, prepuce, scrotum, and shaft. The papers containing these terms and their references were reviewed. Pagetoid dyskeratosis has been observed in lesions on the prepuce and scrotum; this case report now expands the distribution of this finding to the penile shaft. Clinicians and pathologists should be aware of this intriguing potential incidental finding within skin lesions of the male genitalia.

## Introduction

Pagetoid dyskeratosis is a benign incidental pathologic feature. It has been observed in several skin lesions [1]. Herein, a man with penile papules that demonstrated pagetoid dyskeratosis is described, and patients with pagetoid dyskeratosis of the male genitalia are summarized.

## Case presentation

A 26-year-old man with no history of genital dermatoses developed new penile lesions; he was evaluated on several occasions by his primary care physician. The clinical impressions of his lesions included both condyloma acuminata and molluscum contagiosum. On separate occasions, he was treated with either topical imiquimod 5% cream or cryotherapy with liquid nitrogen. Two months later, he noticed new lesions on his penile shaft and sought medical evaluation by a dermatologist.

**Figure 1:**
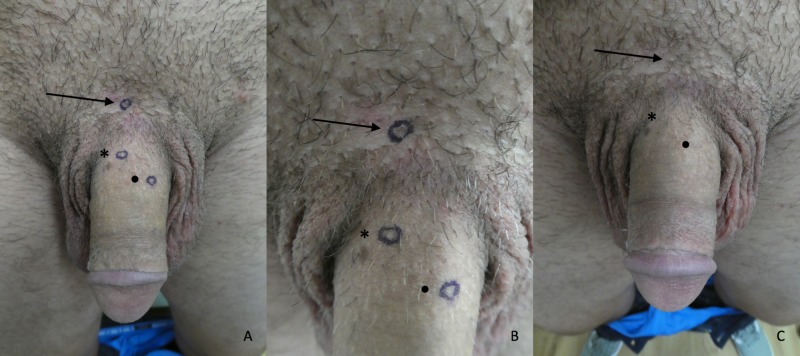
Clinical features of pagetoid dyskeratosis Distant A) and closer B) views with markings and distant C) view without markings show the clinical features of pagetoid dyskeratosis that presented as proximal (arrow), middle (asterisk), and distal (circle) asymptomatic, flesh-colored papules on the proximal dorsal penile shaft of a 26-year-old man.

Clinical examination showed three 1-2 mm asymptomatic, flesh-colored papules located on the proximal portion of the dorsal penile shaft: proximal, middle, and distal, respectively (Figure 1). The site was cleaned with an alcohol swab, the lesions were circled, and lidocaine HCl 1% with epinephrine 1:100,000 was injected locally. The lesions were elevated with Adson forceps and subsequently removed with Metzenbaum scissors. Hemostasis of the biopsy sites was achieved with the application of 20% aluminum chloride. The biopsy sites were treated with topical mupirocin 2% ointment three times daily until the wounds healed.

**Figure 2:**
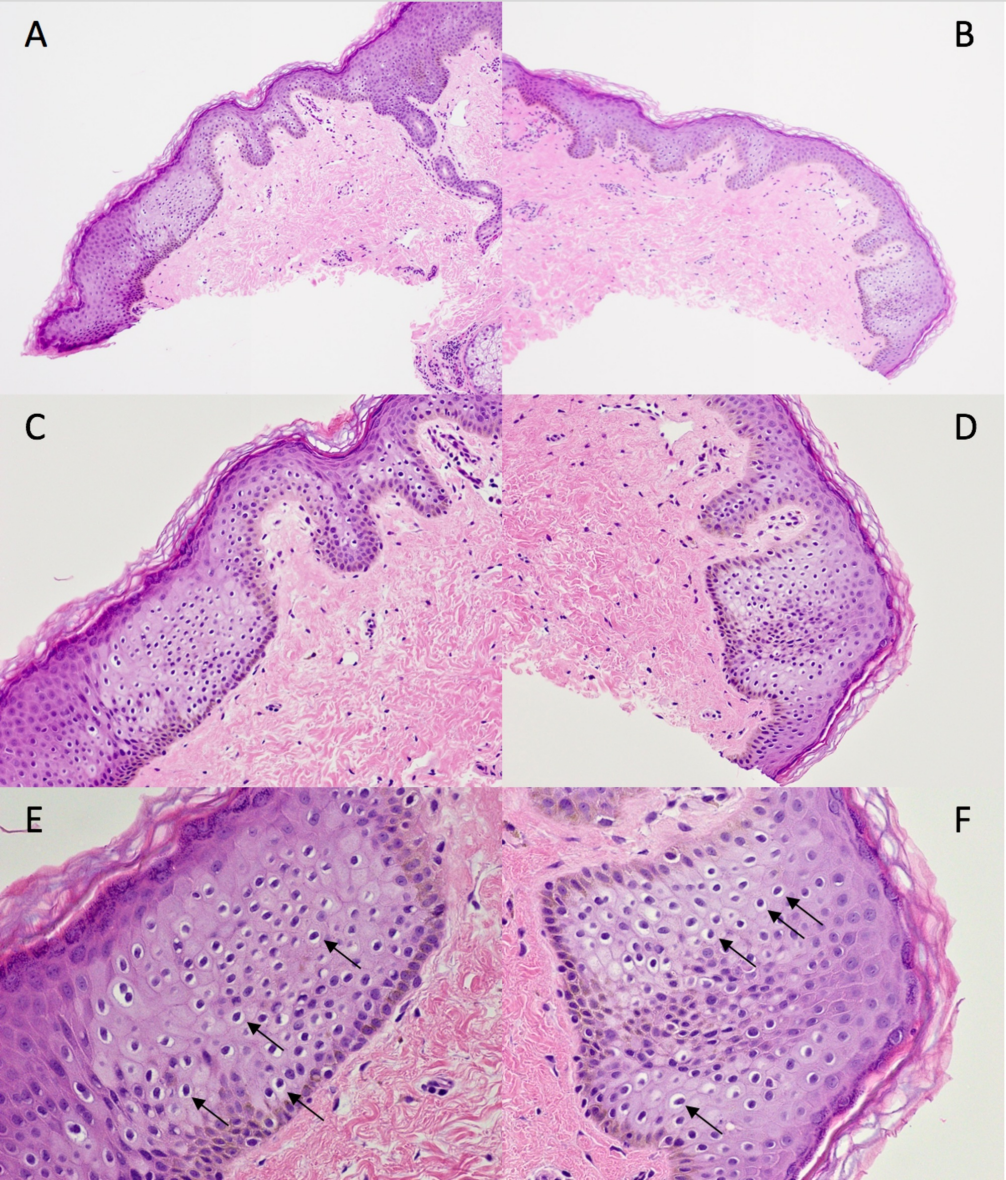
Pathology of the proximal papule Pathology features of pagetoid dyskeratosis are present in the asymptomatic, flesh-colored papule at the base of the dorsal penile shaft of a 26-year-old man. There are multiple large, round intraepidermal pale cells extending singly and in clusters upwards in the epidermis. The pagetoid cells (arrows) contain condensed pyknotic nuclei with perinuclear halos of washed out cytoplasm. Additionally, they demonstrated premature keratinization without acantholysis or parakeratosis. In addition, areas of focal dermal fibrosis were also noted.  (Hematoxylin and eosin: A: x10; B: x10; C: x20; D: x20; E: x40; F: x40).

Microscopic examination was performed; hematoxylin and eosin-stained slides of the lesions were inspected with light microscopy. The most proximal lesion revealed focal dermal fibrosis. In addition, there were multiple large, round intraepidermal pale cells presenting singly and in clusters (Figure 2). The cells resembled those seen in extramammary Paget’s disease, containing condensed pyknotic nuclei with perinuclear halos of clear cytoplasm. Additionally, they demonstrated premature keratinization without acantholysis or parakeratosis.

**Figure 3:**
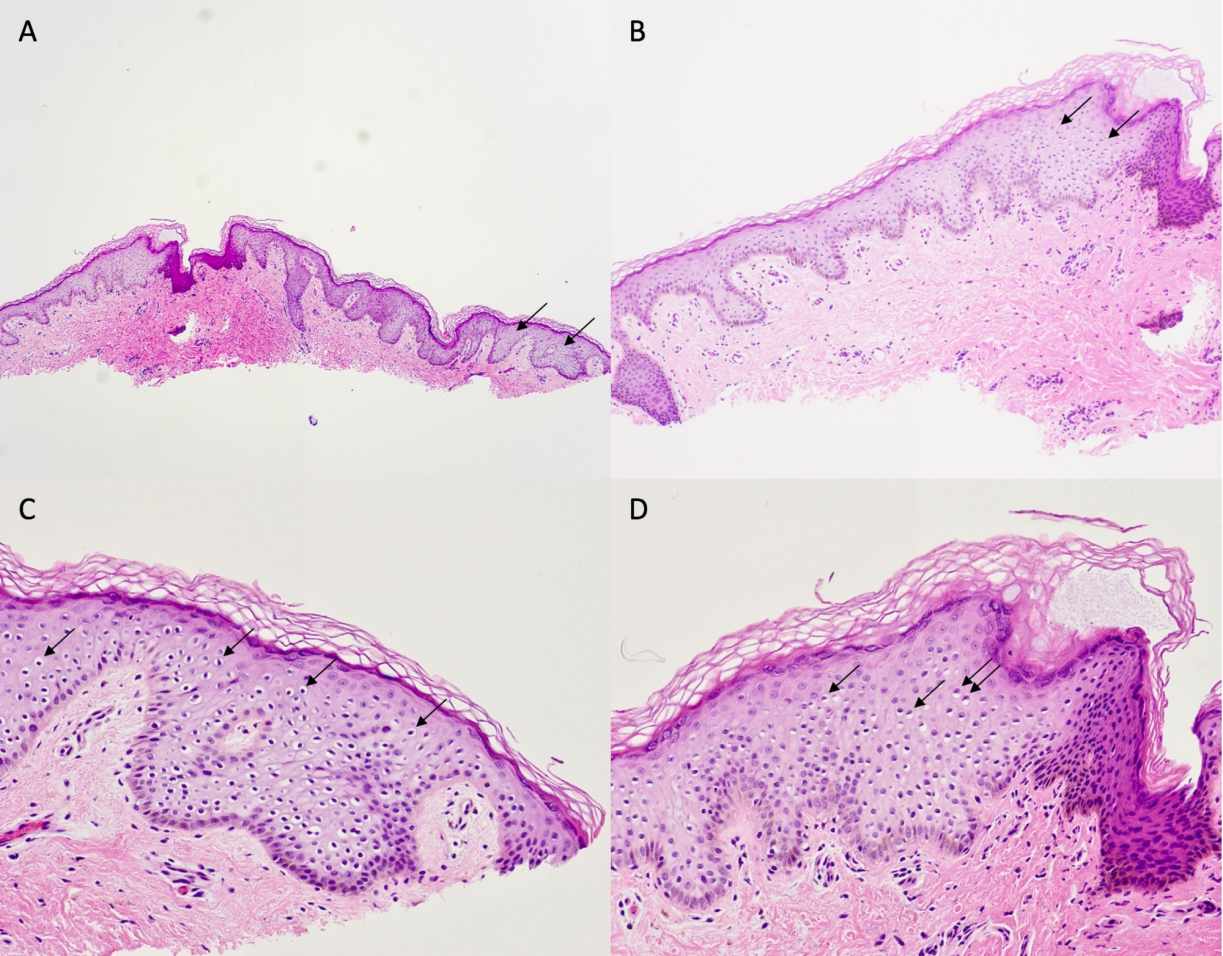
Pathology of the middle papule Pathology features of another asymptomatic, flesh-colored papule on the proximal dorsal penile shaft of a 26-year-old man also demonstrate pagetoid dyskeratosis. The opening of the hair follicle is dilated. Pagetoid cells (arrows) with premature keratinization are also prominent within the epidermis. (Hematoxylin and eosin: A: x4; B: x10; C: x20; D: x20).

The middle lesion revealed a dilated follicular ostium as well as similar changes of pagetoid cells with premature keratinization (Figure 3). The distal lesion only demonstrated sparse superficial dermal fibrosis with mild perifollicular lymphocytic inflammation. The light staining pagetoid cells were not present. Immunoperoxidase staining with p16 was negative within the lesional keratinocytes of all three lesions, making a human papillomavirus infection unlikely.

There has been no recurrence or new lesions in the subsequent six months.

## Discussion

Pagetoid dyskeratosis is an incidental pathologic change. It may occur with several different skin conditions. It is characterized by the presence of intraepidermal keratinocytes that resemble those of extramammary Paget’s disease [1].

This phenomenon was initially interpreted by Mehregan and Civette as artefactual and potentially being related to intraepidermal anesthesia, occlusion, and moisture, or poor sample fixation [2-3]. However, in 1988, Tschen et al. suggested that it more likely represented a process of induced premature keratinization, terming the entity “pagetoid dyskeratosis” since the cells appear similar to those observed in lesions of extramammary Paget’s disease [1]. Subsequently, this pathologic finding has been observed by many other investigators in several distinct anatomic sites and lesions; however, the pathogenesis of pagetoid dyskeratosis remains poorly understood.

The clinical presentation of pagetoid dyskeratosis is variable. It is typically an incidental finding in other primary skin lesions that have been biopsied (Table 1) [1, 4-5]. Hence, the morphology of the lesion-containing pagetoid dyskeratosis corresponds to that lesion’s primary diagnosis.

**Table 1:** Clinical Lesions in Which Pagetoid Dyskeratosis Has Been Observed

Clinical lesion	Diagnostic features
Acrochordon	Pedunculated flesh-colored lesion with a narrow stalk
Actinic cheilitis	Dryness and scaling of the lower lip, often with edema, erythema, and ulceration
Angiofibroma	Red to flesh-colored papules on the face; biopsy shows proliferation of fibroblasts with increased number of blood vessels
Basal cell carcinoma	Pearly nodule with telangiectasias; biopsy shows proliferation of neoplastic basal cells that invade the dermis
Dermal fibrosis	Histopathology shows proliferation of fibroblasts with increased fibrous connective tissue in the dermis
Dermatofibroma	Firm, hyperpigmented nodule which dimples when the adjacent skin is squeezed.
Dilated follicular ostium	Pathology shows widening of the follicular opening
Epidermal inclusion cyst	Cystic, skin-colored, dome-shaped nodule with a central pore
Fibrous papules	Firm, benign, flesh-colored, dome-shaped papule; biopsy shows proliferation of fibroblasts with a fibrotic stroma
Folliculitis	Erythematous, follicular-based pustules
Hemangioma	Red papules; pathology shows a benign proliferation of vessels in the dermis
Irritation fibroma	Small, smooth, pale, pink, benign fibrous tumor of the oral mucosa
Lentigo	Uniformly tan or brown macule with sharp margins; pathology shows hyperpigmentation of the basal layer of the epidermis
Lichen sclerosis	Pruritic, erythematous, atrophic, whitish papules coalescing into plaques; biopsy shows thinning of the epidermis
Melanocytic nevus	Flesh-colored or hyperpigmented, symmetric, uniform, sharply demarcated, round macules, patches, papules, or nodules
Milia	Small white cystic papule caused by keratin retention
Mucocele	Bluish, translucent, fluid-filled papule or nodule of the oral mucosa resulting from chronic irritation
Oral fibrous hyperplasia	Mucosal colored, smooth-surfaced, soft nodular benign mass that may be hyperkeratotic or ulcerated
Oral ulcer	Focal loss of mucosal layer
Scabies	A mineral oil preparation of a skin scraping demonstrates the presence of mites, eggs, or scybala
Seborrheic keratosis	Scaling, tan or brown, greasy papule or plaque with a “stuck-on” appearance
Soft fibroma	Flesh-colored benign tumor composed of fibrous or connective tissue
Squamous cell carcinoma	Nodular, intact or ulcerated, lesion; biopsy shows malignant keratinocytes invading the dermis
Verrucous hyperplasia	Pink papillary exophytic mucosal mass; biopsy shows verrucous projections of hyperplastic epithelium

Pagetoid dyskeratosis is most commonly found in intertriginous areas [1]. However, it can be found nearly anywhere on the body, including the anus (hemorrhoids), buttocks, cervix, extremities, face, hands, nipple, trunk, and vulva [1-2, 5-11]. Pagetoid dyskeratosis of the male genitalia has only been previously described by two other groups of investigators [12-13].

The first study to observe pagetoid dyskeratosis involving the genitalia of men was conducted to examine the histopathology of 281 consecutive patients undergoing circumcision for phimosis. Pagetoid dyskeratosis of the prepuce was incidentally found in 105 individuals (37.4%). The pagetoid cells were most often seen in the superficial layers of the epidermis but were also occasionally present in the parabasal layer [12].

The second report of male genitalia with pagetoid dyskeratosis included a 54-year-old man with a 10-year history of pruritus involving the scrotum. Cutaneous examination of the scrotum revealed mild erythematous and skin-colored patches with focal areas of hyperpigmentation. Histopathologic analysis was consistent with pagetoid dyskeratosis [13].

Our patient had a history of penile shaft and suprapubic lesions that were clinically assessed to be associated with human papillomavirus or pox virus or both. They were treated topically with either imiquimod or liquid nitrogen cryotherapy and resolved. However, he subsequently developed new small papular lesions affecting the previously treated area of his proximal penile shaft that morphologically was not classic in appearance for condyloma acuminatum or molluscum. Indeed, the most prominent feature observed during evaluation of the biopsied lesions was the pagetoid dyskeratosis within the epidermis; the subtle accompanying changes in the dermis (perifollicular inflammation and/or mild dermal fibrosis) correlated with the clinical presentation of the papules.

Microscopically, pagetoid dyskeratosis consists of large round epithelial keratinocytes with pyknotic nuclei and perinuclear halos of washed out cytoplasm. The cells demonstrate premature keratinization into orthokeratotic squames without acantholysis or parakeratosis. They can present individually or in groups often extending upwards in the epidermis. Atypia and mitoses are usually absent [4].

Immunohistochemical staining for high molecular weight cytokeratin and pancytokeratin demonstrates a strong positive signal in the pagetoid cells in comparison to the surrounding keratinocytes [5, 9]. Carcinoembryonic antigen, epithelial membrane antigen, human papillomavirus, and low molecular weight cytokeratin immunohistochemical stains are negative [7]. Staining of pagetoid dyskeratosis cells with alcian blue, Fontana-Masson silver, Mayer’s mucicarmine, Mowry’s colloidal iron, and periodic-acid Schiff typically yields negative results [5, 7].

The pathologic differential diagnosis of pagetoid dyskeratosis includes other conditions with clear cells in the epidermis (Table 2) [3, 14-16]. In some circumstances, immunoperoxidase staining or other stains (in addition to hematoxylin and eosin) may help differentiate these conditions. In our patient, the possibility of condyloma acuminatum was excluded by negative expression of the cells after p16 staining.

**Table 2:** Pathologic Differential Diagnosis of Pagetoid Dyskeratosis CD: cluster of differentiation; CEA: carcinoembryonic antigen; CK: cytokeratin; DEJ: dermal-epidermal junction; EMA: epithelial membrane antigen; HMB: human melanoma black; PAS: periodic acid Schiff; -: negative; +: positive

Pathologic differential diagnosis	Differentiating features
Balloon cell melanoma	Hyperchromatic nuclei surrounded by abundant vacuolated cytoplasm, nests of melanocytes at the DEJ, + S100
Breast carcinoma	Hard, non-mobile, single breast mass with irregular borders; various histologic features depending on type of carcinoma (e.g. ductal, lobular, mixed)
Clear cell acanthoma	Erythematous, sharply demarcated, solitary papule on the lower extremities; basal layer intact, + PAS
Clear cell basal cell carcinoma	Typical features of conventional basal cell carcinoma with clear cells
Clear cell Bowen’s disease (squamous cell carcinoma in situ)	Well-demarcated, erythematous, irregularly bordered plaque with crust or scale; atypia, large nuclei, mitoses, no perinuclear halo, intact basal membrane
Clear cell eccrine carcinoma	Rapidly growing, multinodular dermal neoplasm; ductal differentiation and intracytoplasmic lumen formation, mitoses, prominent nucleoli, + low weight CK, + CEA, + PAS
Clear cell hidradenoma	Granular cells surrounding tubular lumina, hyalinized stroma, + PAS
Clear cell myoepithelioma	Dermal clear cells merging with duct-like structures, + S100, + calponin, + EMA
Clear cell papulosis	White macules and papules distributed along the milk lines; + mucicarmine, + CEA
Clear cell squamous carcinoma	Hydropic degeneration of neoplastic cells, invasion of basal layer, - mucicarmine, - PAS
Clear cell syringoma	Association with diabetes mellitus; nests of eccrine ducts, tadpole-like structures in a fibrous stroma, + PAS
Condyloma acuminatum	Papillated, smooth or soft anogenital papules or plaques; + p16
Extramammary Paget’s disease	Pruritic eczematous, well-demarcated plaque, most often on the vulva; + CK7, + PAS
Langerhans cell histiocytosis	Langerhans cells with “coffee-bean” nuclei, histiocytes, Birbeck granules, + CD1a, + S100
Pagetoid dyskeratosis	Pyknotic nuclei with perinuclear halo, + high weight CKs
Pagetoid melanoma	Neoplastic proliferation of amelanotic melanocytes often in nests, + S100, + HMB45, - PAS, - CK, - CEA
Paget’s disease of the breast	Eczematous changes of the nipple and areola; + mucicarmine, + CEA, + CK7
Renal cell carcinoma	Flank mass, hematuria, paraneoplastic syndromes; lipid and glycogen-rich cytoplasm
Sebaceous adenoma	Benign proliferation of sebaceous cells
Sebaceous carcinoma	Eyelid lesion; atypia, mix of undifferentiated and sebaceous cells, scalloping of the nuclei
Sebaceous epithelioma	Malignant proliferation of irregularly shaped cells, half of which demonstrate sebaceous differentiation
Superficial spreading malignant melanoma	Irregularly bordered, multi-colored, pigmented plaque; atypical, hyperchromatic, neoplastic melanocytes that lack cellular maturation, + S100, + HMB45
Toker cell of the nipple	Found in the nipple epidermis of some normal women; - mucicarmine, + CK7
Tricholemmal carcinoma	Atypias, mitoses, + PAS
Tricholemmoma	Lobular growth of clear cells around hair follicles, peripheral palisading, + PAS

The pathogenesis of pagetoid dyskeratosis remains to be definitively established. Tschen et al. hypothesized that the cells represent a small population of the normal keratinocytes that are induced to proliferate by an external trigger, such as mechanical trauma or friction [1]. Piqué-Duran et al. found that lesions of the axilla more frequently demonstrated pagetoid dyskeratosis in comparison to those of other locations; they suggested that this observation supports the theory that moisture and friction contribute to the development of pagetoid dyskeratosis [14].

Pagetoid dyskeratosis is an incidental finding. Typically, there are no clinical sequelae. Therefore, management is directed toward the primary lesion.

## Conclusions

Pagetoid dyskeratosis is an intriguing benign pathologic feature. It has been observed in a variety of cutaneous lesions located on various areas of the body. Pagetoid dyskeratosis of the male genitalia is uncommon and has been previously described on either the scrotum or the prepuce; our patient’s lesions were on the proximal penile shaft. The differential diagnosis of pagetoid dyskeratosis includes other conditions characterized by clear cells in the epidermis. In particular, in men with genital lesions, the differential diagnosis includes venereal warts. This was excluded based on microscopic findings and negative p16 staining. Pagetoid dyskeratosis-directed therapy is usually not necessary and management of the patient is based upon treating the primary skin lesion.
